# Conflicts of interest among committee members in the National Academies’ genetically engineered crop study

**DOI:** 10.1371/journal.pone.0172317

**Published:** 2017-02-28

**Authors:** Sheldon Krimsky, Tim Schwab

**Affiliations:** 1 Department of Urban & Environmental Policy & Planning, Tufts University, Medford, Massachusetts, United States of America; 2 Food & Water Watch, Washington, DC, United States of America; the University of Sydney, AUSTRALIA

## Abstract

The National Academies of Sciences, Engineering and Medicine (NASEM) publishes numerous reports each year that are received with high esteem by the scientific community and public policy makers. The NASEM has internal standards for selecting committee members that author its reports, mostly from academia, and vetting conflicts of interest. This study examines whether there were any financial conflicts of interest (COIs) among the twenty invited committee members who wrote the 2016 report on genetically engineered (GE) crops. Our results showed that six panel members had one or more reportable financial COIs, none of which were disclosed in the report. We also report on institutional COIs held by the NASEM related to the report. The difference between our findings and the NASEM reporting standards are discussed.

## Introduction

During the mid-1980s, the first journals introduced requirements that authors disclose any financial interests they have on the subject matter of their submitted papers. By the early 1990s, the world’s leading science journals and the majority of medical journals had adopted conflict of interest (COI) policies for authors. Soon thereafter, COI policies were established by the National Science Foundation and the Public Health Service for their grant recipients.

Financial COI disclosures by individuals serving on scientific panels responsible for developing guidelines, such as clinical practice guidelines [1], or writing reports on behalf of professional organizations became another arena for transparency of financial COIs. After a study was published highlighting the undisclosed conflicts of interest of panel members who established guidelines in the Diagnostic and Statistical Manual of Mental Illness (DSM-IV), considerable media attention was directed at the American Psychiatric Association, publisher of the DSM volumes, calling for greater transparency [2]. In the next edition of the DSM (DSM-5), conflict of interest disclosures by DSM-5 panel members were cited in the volume.

Disclosure of conflicts of interest by individuals and institutions has become a widely accepted ethical norm and a legal requirement in science and medicine. Failure to disclose medical financial conflicts of interest has even been grounds for a lawsuit against a prominent university [3].

The growth of COI disclosure policies for university researchers was influenced by a body of social science research that discovered a new effect, termed the “funding effect” [4, 5]. This finding showed that studies funded by private companies, compared to independent non-profit and government sources as controls, tended to produce outcomes consistent with the financial interest of those companies [6,7]. The effect can be found in data collection and in the interpretation of results.

The “funding effect” has been reported across a number of fields, including pharmaceuticals [8], chemical toxicology [9], tobacco [10], surgery [11], mobile phones [12], nutrition [13], and biotechnology. Diels et al. (2011) examined research on genetically modified crops and found that “COIs through professional affiliations or direct research funding are likely to influence the final outcome of such studies in the commercial interest of the involved industry [14]. Another meta-analysis documented widespread conflicts of interest in published research about efficacy and durability of genetically engineered crops, and also found that the presence of conflicts was associated with favorable outcomes to agricultural biotechnology companies [15].

### Federal advisory bodies

The federal government had long established conflict of interest requirements for its employees and for members of Congress, but scientists and experts serving on federal advisory committees were not covered under those regulations prior to 1972 when the Federal Advisory Committee Act (FACA) was passed. Under FACA, scientists invited to serve on federal advisory committees are prohibited from holding substantial conflicts of interest on matters before the committee on which they serve. This applied to all federal agencies including the Food and Drug Administration, the National Institutes of Health and the Centers for Disease Control.

The Federal Advisory Committee Act set certain principles for government agencies when setting up advisory committees. The act stipulated that balance, independence and transparency were requirements for establishing a federal advisory committee. The General Services Administration (GSA) was given the responsibility for developing regulations and guidance for federal agencies that establish advisory committees under FACA and the Office of Government Ethics (OGE) was responsible for issuing regulations on conflicts of interest for advisory committee members (see 18 U.S.C **§**208). In addition to the requirements of GSA and OGE, federal agency heads have responsibility for issuing guidelines and management procedures for addressing conflicts of interest on advisory committees they appoint. Members who serve on federal advisory committees are defined as special government employees, appointed by a government agency to perform certain temporary duties, with or without compensation [16].

### The national academies

The NASEM, a private, non-profit institution chartered by Congress, has always functioned as an advisory body to federal agencies and Congress since it was established in 1863. It recruits scientists from academia, industry and non-profit organizations, who serve on an unpaid basis to author studies and provide technical consultation to the government. Nevertheless, the organization did not fall under the Federal Advisory Committee Act in 1972.

The Academies receive hundreds of millions of dollars in annual contributions from private and public sources [17], and it has long maintained close ties to industry that have raised conflicts-of-interest allegations—at times directed at the committees of invited scientists that author its reports [18]. A 1970 committee examining the health effects of lead came under scrutiny because several committee members were employed by companies producing lead additives [19]. Shortly after, a committee examining food chemicals and cancer drew controversy because some members were consultants to the food industry, which was also providing funding to the committee [19].

In amendments made to FACA in 1997, specific COI requirements were laid out for committees of the NASEM, at that time called the National Academy of Sciences [20]. Congress noted that federal agencies could not utilize the scientific advice of the Academy unless the following instructions were heeded:

1. no individual appointed to serve on the committee has a conflict of interest that is relevant to the functions to be performed, unless such conflict is promptly and publicly disclosed and the Academy determines that the conflict is unavoidable,
2. the committee membership is fairly balanced as determined by the Academy to be appropriate for the functions to be performed [21].

The NASEM, noting its requirements under FACA, has developed its own conflicts-of-interest guidelines that define a conflict as “any financial or other interest which conflicts with the service of the individual because it (1) could significantly impair the individual’s objectivity or (2) could create an unfair competitive advantage for any person or organization” [22]. The interest in question must be closely related to the work of the committee.

According to the NASEM, removing financial conflicts eliminates potentially compromising situations in which others “could reasonably question, and perhaps discount or dismiss, the work of the committee simply because of the existence of such conflicting interests” [22].

This language appears in line with a National Academies’ guidebook of best practices for scientists, “On Being a Scientist” (now in its third edition), which notes that “even the appearance of a financial conflict of interest can seriously harm a researcher’s reputation as well as public perceptions of science” [23].

Despite its attention to conflicts of interest, The Academies’ work continues to raise questions, stemming, in part, from the continued recruitment of scientists who have financial interests in the field studied by committees on which they serve [18]. By 1998, the year after FACA was amended to include The Academies, twenty-four percent of their committee members worked for industry [24]. A 2006 investigation by a public-interest group examined twenty-one NASEM committees, finding that nearly one in five committee members had financial ties to industry [25,26].

Under FACA, the NASEM may appoint scientists with financial COIs, but must make every effort to avoid doing so—and may only do so when it is an “unavoidable” conflict of interest. If the NASEM appoints a committee member with a financial COI, it is obligated to “promptly and publicly” disclose it. Critics have argued that the NASEM does not follow FACA guidelines by virtue of the number of panel members with financial COIs, often undisclosed.

One area of the NASEM’s work that has repeatedly drawn criticism around conflicts of interests over the last two decades concerns agricultural biotechnology, as numerous media outlets have reported on a variety of alleged conflicts related to several reports [27–29]. In 2016, the NASEM issued one of its most comprehensive studies on GE agriculture, a topic that has drawn considerable public debate. Because of the importance of this study and the fact that the NASEM has long had a conflicts-of-interest policy in place, we chose to examine this report.

### Goals and methods of the study

In our study we examine financial COIs among committee members of the NASEM publication issued in May 2016 titled *Genetically Engineered Crops*: *Experiences and Prospects* (hereafter the NASEM2016 report). One of the principal objectives of the NASEM2016 report was to assess the “basis of evidence for purported negative effects of GE crops and their accompanying technologies, such as poor yields, deleterious effects on human and animal health, increased use of pesticides and herbicides, the creation of ‘super-weeds,’ reduced genetic diversity, fewer seed choices for producers, and negative impacts on farmers in developing countries and on producers of non-GE crops, and others, as appropriate” [30].

In our study we sought to determine whether there were undisclosed financial COIsamong committee members in the NASEM2016 report. We compared our results on financial COIs with the NASEM’s guidelines and its recommendations on how conflicts of interest among members of its committees are handled.

We restricted our analysis to financial COIs—and disregarded other conflicts such as conflicts of commitment or ideological and political positions held by committee members—because an analysis of financial COIs allowed us to draw on well-established criteria from the NASEM and a variety of other guidelines.

We examined the conflicts of interest of twenty committee members who developed, wrote, and approved the analysis and made recommendations in the

NASEM2016 report. We applied a multi-modal method of ascertaining whether panel members held a financial conflict of interest that involved examining funding sources, patents, company advisory boards and consulting [31].

The following criteria were used to determine the existence of a financial COI. If within three years prior to the start of the study in 2014, a panel member was found to have one or more of the following financial interests related to the subject matter of the report, such findings were recorded as a financial COI for that panel member: (1) holds a patent or patent application on a genetically modified crop or a process involved in producing genetically modified crops. (2) holds equity in a company with a financial interest in the success or failure of genetically engineered (GE) crops; (3) serves or served on an advisory committee of such a company; (4) received research funding, in-kind contributions, or research supplies (other than donated seeds) for work related to GE crops from such a company; (5) employed by such a company, or by a non-profit that is primarily funded by such a company; (6) consults for such a company.

These six categories are found in the NASEM’s own definitions of financial COIs, however our criteria differs from the NASEM in two ways.

First, the NASEM only considers financial investments, i.e. equity holdings, greater than $10,000 to represents a disclosable financial COI, whereas we placed no threshold on financial investments [22].

The U.S. Department of Health and Human Services, which oversees the National Institutes of Health, recently reduced its reporting threshold from $10,000 to $5,000 [32]. The National Academy of Medicine, in recommendations it issued for biomedical institutions in 2009, determined that no thresholds should be used in financial COI forms, and that all financial relationships should be disclosed for review [33]. It also noted that “Because bias is unintentional and not a matter of corruption, however, small gifts may still produce results and therefore should not be assumed to be benign.” Given the scientific literature showing that the size of a financial relationship may be irrelevant to whether it creates bias, we chose to put no threshold on the value of a committee member’s financial investments [34].

Second, our criteria may also differ from the NASEM’s in the scope of our financial COI review. The NASEM only examines committee members’ “current interests,” noting that it “does not apply to past interests that have expired, no longer exist, and cannot reasonably affect current behavior” [30]. This language is included in the instructions given to committee members on financial COI disclosure forms, who apparently must interpret for themselves whether any of their previous financial relationships could “reasonably affect current behavior” [35].

A number of contemporary guidelines recognize that previous financial relationships can reasonably affect judgment and sometimes require five years of financial COI reporting [36]. The International Committee of Medical Journal Editors (ICMJE) uses three years as the disclosure interval for authors submitting publications to journals [37]. The American Psychiatric Association requires disclosure covering a period of the last three calendar years and the current year [38]. The National Academy of Medicine in 2009 noted that scientific institutions typically review any financial COIs from the previous year, and sometimes review as many as five years of information [39]. We chose a three-year review period.

Holding a patent or patent application that is active during one’s tenure on the NASEM2016 committee was sufficient to meet our criteria and also appears to meet the NASEM’s “current interests” standard. However, we cannot be confident that our 3-year reporting period for other categories of financial interests—such as previously having consulted for or received research funding from a company—is what the NASEM and committee members interpreted as a previous financial COI that could “reasonably affect” judgment.

The NASEM2016 study began in late 2014, so we examined financial COIs of committee members starting in 2012. Each of the twenty listed committee members was evaluated for financial conflicts of interest from 2012–2016 through reviewing published studies in journals with COI disclosures, committee members’ online biographies or curriculum vitae, databases that list scientist-industry collaborations, news articles that highlight scientists’ company activities, and the United States Patent and Trademark Office’s patent database.

### Institutional conflict of interest at the academies

Though much of the literature and discussion surrounding the funding effect concerns individual researchers, there is now a growing appreciation of the potential bias introduced through institutional conflicts of interest (institutional COIs). The National Academy of Medicine notes that an institutional COI exists when an “institution’s own financial interests or those of its senior officials pose risks of undue influence on decisions involving the institution’s primary interests” [40].

The Association of American Medical Colleges and American Association of Universities have issued guidelines for human-subject research that enumerate sources of potential institutional COIs, which include “substantial gifts (including gifts in kind) from a potential commercial sponsor” [41]. These guidelines also recommend that institutional leaders establish an organizational culture around conflicts of interest, “demonstrat[ing] to the academic community and to the public that compliance with these policies, including full disclosure of financial conflicts of interest, is an imperative reflecting core institutional values.” The American Association of Universities makes the very broad, unqualified recommendation around institutional COIs to “disclose always” [42].

The NASEM’s conflicts-of-interest policy does not address institutional COIs, and it is not known if or how The Academies addresses this issue. Nevertheless, because the NASEM is a private institution, which receives funding from private entities that may have a financial interest in the NASEM’s work, we examined the role that private entities play in the NASEM’s operations to determine whether institutional COIs exist and if or how they were disclosed.

### Findings

The NASEM 2016 report discusses its finding that no committee members had financial conflicts of interest.

“Every Academies committee is provisional until the appointed members have had an opportunity to discuss as a group their points of view and any potential conflicts of interest related to the statement of task. They also determine whether the committee is missing expertise that may be necessary to answer questions in the statement of task. As part of their discussion, committee members consider comments submitted by the public about the committee’s composition. The discussion takes place in the first in-person meeting of the committee. The committee is no longer provisional when it has determined that no one with an avoidable conflict of interest is serving on the committee and that its membership has the necessary expertise to address the statement of task” [30].

The report stated, without qualification, that the NASEM “did not identify” any conflicts of interest among the twenty panel members. “The Committee on Genetically Engineered Crops did not identify any conflicts of interest among its members. However, in light of comments received from the public before its first meeting and because of two resignations around the time of the first meeting, one new member with experience in molecular biology and two new members with international experience and expertise in sociology were added to the committee. Those appointments brought the committee’s membership to 20. That is a large committee for the Academies, but it ensured that diverse perspectives were represented in committee discussions and in the final report” [30].

By contrast, our inquiry found that six out of twenty committee members had financial COIs (Table 1). Five individuals received research funding from for-profit companies related to the subject matter of the report and five had patents or patent applications on the subject matter of GE crops. Four panel members had two financial COIs. In total there were ten financial COIs among the six committee members, and it would appear that most of these conflicts meet the NASEM’s own standards for financial COIs.

**Table 1:** Financial Conflicts of Interest of NASEM Panel Members: *Committee on Genetically Engineered Crops*: *Past Experience and Future Prospects*.

Committee Member	AEH	BAC	CRF	DPPA	EEMP	FCON
Fred Gould, North Carolina State University, Raleigh, NC						
Richard M. Amasino, University of Wisconsin–Madison, Madison, WI				X2-D		
Dominique Brossard, University of Wisconsin-Madison, Madison, WI						
C. Robin Buell, Michigan State University, East Lansing, MI			X4-C	X 4-D		
Richard A. Dixon, University of North Texas, Denton,TX			X5-C	X5-D		
Josė B. Falck-Zepeda, International Food Policy Research Institute (IFPRI), Washington, DC						
Michael A. Gallo, Rutgers-Robert Wood Johnson Medical School (retired), Piscataway, NJ						
Ken Giller, Wageningen University, Wageningen, The Netherlands						
Leland Glenna, Pennsylvania State University, University Park, PA						
Timothy S. Griffin, Tufts University, Medford, MA						
Bruce R. Hamaker, Purdue University, West Lafayette, IN						
Peter M. Kareiva, The Nature ConservancyWashington, DC						
Daniel Magraw, Johns Hopkins University School of Advanced International Studies, Washington, DC						
Carol Mallory-Smith, Oregon State University, Corvallis, OR			X14-C			
Kevin Pixley, International Maize and Wheat Improvement Center (CIMMYT), Texcoco, Mexico						
Elizabeth P. Ransom, University of Richmond, Richmond, VA						
Michael Rodemeyer, University of Virginia (formerly), Charlottesville, VA						
David M. Stelly, Texas A&M University and Texas A&M AgriLife Research, College Station, TX			X18-C	X18-D		
C. Neal Stewart, University of Tennessee, Knoxville, TN			X19-C	X19-D		
Robert J. Whitaker, Produce Marketing Association, Newark, DE						

EH = equity holding; AC = advisory committee; RF = research funding; PPA = patent or patent application; EMP = employed by a company; CON = consulting for a company.

Five of the six committee members with financial COIs had patents or industry research funding during the time that they served on the NASEM2016 study, which would appear to meet the NASEM’s standard for “current” financial COIs. The only committee member who may not fall under the NASEM’s disclosure criteria was Carol Mallory-Smith, who reported receiving industry research funding in a 2012 journal article. This funding falls within our three-year window, but did not occur while Mallory-Smith served on the NASEM2016 committee and may or may not have met the NASEM’s criteria for a financial COIs.

In all cases, the financial COIs concerned private interests that have a financial interest in the promotion of genetic engineering, not in opposition to it.

Table 1 shows the range of financial COIs found for the panel members and their sources and Table 2 provides the documentation for the financial COI determination.

**Table 2:** Sources of Financial COIs of NASEM Committee Members.

Committee Member	Sources of Financial Conflicts of Interests
**Amasino**	**2-D** Amasino RM, Gan S, Hoh Y, inventors; Wisconsin Alumni Research Foundation, assignee. Transgenic plants with altered senescence characteristics. United States patent 6,359,197. 2002 Mar 9. Available: http://patft.uspto.gov/netacgi/nph-Parser?Sect1=PTO2&Sect2=HITOFF&p=1&u=%2Fnetahtml%2FPTO%2Fsearch-bool.html&r=9&f=G&l=50&co1=AND&d=PTXT&s1=amasino.INNM.&s2=transgenic&OS=IN/amasino+AND+transgenic&RS=I/amasino+AND+transgenic. Accessed 2016 Aug 24. Amasino RM, Gan S, inventors; Wisconsin Alumni Research Foundation, assignee. Transgenic plants with altered senescence characteristics. United States patent 5,689,042. 2002 Mar 19. Available: http://patft.uspto.gov/netacgi/nph-Parser?Sect1=PTO2&Sect2=HITOFF&p=1&u=%2- Fnetahtml%2FPTO%2Fsearch-bool.html&r=10&f=G&l=50&co1=AND&d=PTXT&s1=amasino.- INNM.&s2=transgenic&OS=IN/amasino+AND+transgenic&RS=IN/amasino+AND+transgenic. Accessed 2016 Aug 24.
**Buell**	**4-C** Andrew T. Wiersma, Todd A. Gaines, Christopher Preston, John P. Hamilton, Darci Giacomini, C. Robin Buell, Jan E. Leach, Philip Westra. Gene amplification of 5-enol-pyruvylshikimate-3-phosphate synthase in glyphosate-resistant Kochia scoparia. Planta (2015) 241:463–474. DOI 10.1007/s00425-014-2197-9
	**4-D** Collmer A, Alfano JR, Tang X, Buell CR, Martin GB, inventors; Cornell Research Foundation, Inc. et al, assignees. Nucleic acids encoding pseudomonas hop proteins and use thereof. United States patent 7,138,569. 2006 November 21. Available: http://patft.uspto.gov/netacgi/nph-Parser?Sect1=PTO2&Sect2=HITOFF&p=1&u=%2Fnetahtml%2FPTO%2Fsearch-bool.html&r=3&f=G&l=50&co1=AND&d=PTXT&s1=collmer.INNM.&s2=nucleic&OS=IN/collmer+AND+nucleic&RS=IN/collmer+AND+nucleic. Accessed 2016 Aug 24.
**Dixon**	**5-C** Dixon R. University of North Texas Faculty Information System online database. Available https://facultyinfo.unt.edu/faculty-profile?query=Richard+Dixon&type=name&profile=rad0169. Accessed 2016 Aug 24. Gallego-Giraldo L, Shadle G, Shen H, Barros-Rios J, Corrales SF, Want H et al. Combining enhanced biomass density level for improved forage quality. Plant Biotechnol J. 2016 Mar;14(3):895–904 Available : http://onlinelibrary.wiley.com/doi/10.1111/pbi.12439/epdf. Accessed 2016 Aug 24.
	**5-D** Zhao Q, Chen F, Dixon, R, inventors ; The Samuel Roberts Nobel Foundation, assignee. Plants with modified lignin content and methods for production thereof United States patent 8,796,509. 2014 Aug 5. Available: http://patft.uspto.gov/netacgi/nph-Parser?Sect1=PTO2&Sect2=HITOFF&p=1&u=%2Fnetahtml%2FPTO%2Fsearch-bool.html&r=9&f=G&l=50&co1=AND&d=PTXT&s1=%22Dixon,+Richard%22.INNM.&OS=IN/%22Dixon,+Richard%22&RS=IN/%22Dixon,+Richard%22. Accessed 2016 Aug 24.
	Dixon, R, Chen F, Wang Z, inventors ; The Samuel Roberts Nobel Foundation, Inc., assignee. Biofuel production methods and compositions. United States patent 9,309,528. 2016 Apr 12. Available: http://patft.uspto.gov/netacgi/nph-Parser?Sect1=PTO2&Sect2=HITOFF&p=1&u=% 2Fnetahtml%2 FPTO%2Fsearch-bool.html&r=2&f=G&l=50&co1=AND&d=PTXT&s1=%22Dixon,+Richard%22. INNM.&OS=IN/%22Dixon,+Richard%22&RS=IN/%22Dixon,+Richard%22. Accessed 2016 Aug 24. Li W, Uppalapati SR, Mysore KS, Dixon RA, inventors ; The Samuel Roberts Nobel Foundation, Inc., assignee. Metabolic engineering for plant disease resistance. United States patent 9,238,821. 2016 January 19. Available : http://patft.uspto.gov/netacgi/nph-Parser?Sect1=PTO2&Sect2=HITOFF&p=1 &u=%2Fnetahtml%2FPTO%2Fsearch-bool.html&r=3&f=G&l=50&co1=AND&d=PTXT &s1=%22Dixon,+Richard%22.INNM.&OS=IN/%22Dixon,+Richard%22&RS=IN/%22Dixon,+Richard%22. Accessed 2016 Aug 24. Shen H, Chen F, Dixon RA, inventors; The Samuel Roberts Noble Foundation, Inc., assignee. Compositions and methods for improved plant feedstock. United States patent 8,901,371. 2014 Dec 2. Available : http://patft.uspto.gov/netacgi/nph-Parser?Sect1=PTO2&Sect2=HITOFF&p=1&u=%2Fnetahtml%2FPTO%2Fsearch-bool.html&r=7&f=G&l=50&co1=AND&d=PTXT&s1=%22Dixon,+Richard%22.INNM.&OS=IN/%22Dixon,+Richard%22&RS=IN/%22Dixon,+Richard%22. Accessed 2016 Aug 24. SEE List of Additional Patents at: Dixon R. University of North Texas Faculty Information System online database. Available https://facultyinfo.unt.edu/faculty-profile?query=Richard+ Dixon&type=name&profile=rad0169. Accessed 2016 Aug 24.
**Mallory-Smith**	**14-C** Zapiola M, Mallory-Smith CA. Crossing the divide: gene flow produces intergeneric hybrid in feral transgenic creeping bentgrass population. http://onlinelibrary.wiley.com/doi/10.1111/j.1365-294X.2012.05627.x/abstract? systemMessage=Wiley+Online+Library+will+be+unavailable+on+Saturday+3rd+September+2016+at+08.30+BST%2F+03%3A30+EDT%2F+15%3A30+SGT+for+5+hours+and+Sunday+4th+September+at+10%3A00+BST%2F+05%3A00+EST%2F+17%3A00+SGT+for+1+hour++for+essential+maintenance.+Apologies+for+the+inconvenience. Accessed 2016 Aug 24.“Support also was received from special grants from USDA-Animal and Plant Health Inspection Service (APHIS) and The Scotts Company.”
**Stelly**	**18-C** Hulse-Kemp AM, Lemm J, Plieske J, Ashrafi H, Buyyarapu R, Fang DD et al. Development of a 63K SNP Array for Cotton and high-density mapping of intra- and inter-specific populations of Gossypium spp. G3 (Bethesda). 2015 Apr 22;5(6):1187–209. Available: http://www.g3journal.org/content/early/2015/04/22/g3.115.018416.full.pdf+html. Accessed 2016 Aug 24. Zhang T, Hu Y, Jiang W, Fang L, Guan X, Chen J et al. Sequencing of allotetraploid cotton (Gossypium hirsutum L. acc. TM-1) provides a resource for fiber improvement. Nat Biotechnol.) 2015 May;33(5):531–7. Available: http://www.nature.com/nbt/journal/v33/n5/full/nbt.3207.html. Accessed 2016 Aug 24.
	**18-D.** Rooney W, Hodnett GL, Kuhlman LC, Stelly DM, Price HJ, inventors; The Texas A&M University System, assignee. Intergeneric hybrid plants and methods for production thereof. United States patent 8,362,329. 2013 January 29. Available: http://patft.uspto.gov/netacgi/nph-Parser?Sect1=PTO2&Sect2=HITOFF&p=1&u=%2Fnetahtml%2FPTO%2Fsearch-bool.html&r=2&f=G&l=50&co1=AND&d=PTXT&s1=stelly.INNM.&s2=david.INNM.&OS=IN/stelly+AND+IN/david&RS=IN/stelly+AND+IN/david. Accessed 2016 Aug 24.
**Stewart**	**19-C** Stewart CN. University of Tennessee. C.V. Available: http://plantsciences.utk.edu/pdf/stewart%20_cv_%202015_%20midyear_public_long.pdf. At 18–21. Accessed 2016 Aug 24. Peng Y, Lai Z, Lane T, Nageswara-Rao M, Okada M, Jasieniuk M. De Novo Genome Assembly of the Economically Important Weed Horseweed using Integrated Data from Multiple Sequencing Platforms. Plant Physiology. November 2014 vol. 166 no. 3. Available: http://www.plantphysiol.org/content/166/3/1241.full Accessed 2016 Aug 24.
	**19-D** Stewart CN. University of Tennessee. C.V. Available: http://plantsciences.utk.edu/pdf/stewart%20_cv_%202015_%20midyear_public_long.pdf. At 16–17. Accessed 2016 Aug 24.

N (1–20) from Table 1 indicates the committee member. A-F indicates the type of financial COI. A = equity holding (EH); B = advisory committee (AC); C = research funding (RF); D = patent or patent application (PPA); E = employed by company (EMP); F = consulting for a company (CON).

### Institutional COI findings

Just as the NASEM did not disclose any financial COIs among its committee members, it also did not disclose institutional COIs. At the time the NASEM was developing its 2016 GE crop report, it was receiving money from agricultural biotechnology companies that have a financial interest in the study. The organization’s annual financial reports do not give exact figures but note that three leading agricultural biotechnology companies (Monsanto, Dupont and Dow) have given up to $5 million dollars each to the NASEM [43]. Some of the companies’ donations have been directed to NASEM projects focused on agricultural biotechnology. This includes a 2015 NASEM workshop on how to communicate the science about GE agriculture to members of the public, which was co-organized by the committee chair of the NASEM2016 report [44]. The NASEM did not disclose this funding on the printed agenda handed out at this workshop, but months later issued a report that did disclose the funding [44–45].

The NASEM also disclosed the funders of the NASEM2016 report—which came from foundations and the USDA—but the NASEM failed to disclose the fact that it has received millions of dollars in institutional support from agricultural biotechnology companies.

Another potential institutional COI relates to the institutions boards at the NASEM that oversee research projects on genetic engineering, which have long included representatives from companies with a financial interest in the outcome of these studies [46–48]. The NASEM Board on Agriculture and Natural Resources, which helped oversee the NASEM2016 report, included a representative from Monsanto and representatives from several other food and feed companies including Cargill, Nestle Purina and Novus International [30].

The front matter of the NASEM2016 report disclosed this, but it is unclear how The Academies managed these potential institutional COIs. The Association of Governing Boards of Colleges and Universities has addressed such institutional COIs by asking board members to recuse themselves from matters in which they have a financial interest [49].

## Discussion

Our finding that the NASEM failed to disclose financial COIs held by committee members raises questions about The Academies’ implementation of the FACA requirements and the effectiveness of its financial COI policy. Whether this is due to weak enforcement of financial COI policies by the NASEM, a failure of committee members to disclose financial COIs to the NASEM, weaknesses within the language of FACA, or other explanations is a subject of further inquiry.

Congressional language in FACA gives The Academies substantial discretion to assess and manage financial COIs. As stated in the legislation, “The Academy shall make its best efforts to insure” that financial conflicts of interest are avoided, or disclosed if unavoidable, and that committees are balanced [21].

The NASEM maintains an official conflicts-of-interest policy, which speaks to its requirements under FACA, and all committee members are required to submit a form about financial COIs and other “background information” [50]. The form asks a broad range of questions about committee members’ professional work related to the subject matter of the study, including any public statements they have made, and financial COIs, including investments, research funding, patents, consulting and several other activities [22]. These disclosures are confidential and not available for public review.

Potential weaknesses in the NASEM’s financial COI review include, as noted previously, its high threshold for financial investments ($10,000 or greater). Additionally, the NASEM’s disclosure forms instruct committee members to focus on disclosing “current” financial COIs, which may not capture recent financial relationships that could reasonably affect judgment.

Even if committee members did not disclose all of the financial COIs we documented, The Academies were apprised of conflicts from other sources. When the NASEM undertakes a study, it allows stakeholders to submit public comments. Under FACA requirements, the NASEM is supposed to make the public comments it receives available for public review, [21] but the NASEM informed us that it does not allow public review of comments related to committee composition, including those that allege financial COIs. (William J. Skane, Email, 2016 October 16).

Nevertheless, we were able to secure and review several letters submitted to the NASEM that discuss problems with the committee. One letter, co-signed by 23 individuals, mostly from academia, criticized the committee composition, noting that “the panel includes staff and representatives from institutions and agencies that have an established history and institutional orientation toward seeking technological approaches to intensifying agricultural production and prioritizing yield increases over addressing the complex amalgam of factors that contribute to achieving authentic food security” and that this makes it “more challenging for those committee members to bring an independent, critical eye to their assessment project” [51]. Another letter to The Academies provided documentation alleging financial COIs of numerous committee members, including the patent interests and research funding of two committee members identified in our financial COI analysis [52].

The final authority on financial COI decisions for NASEM reports rests with the chair of the National Research Council, the research arm of the NASEM [22]. However, an additional and apparently integral part of the NASEM review process includes committee members openly discussing amongst themselves issues of balance and financial COIs. The NASEM notes that at the first committee meeting, committee members, who are still considered to be “provisional,” “are asked to discuss the issues of committee composition and balance and conflict of interest, and the relevant circumstances of the individual members…”[22].

Professional colleagues may be reluctant to candidly and openly judge each other’s fitness to serve on the committee, however, especially around a sensitive subject like financial COIs. Likewise, it is unclear why the NASEM waits until after the committee has been announced and held its first public meeting to finalize its financial COI reviews, as this puts The Academies in a potentially awkward position of having to publicly announce one or more “provisional” committee members being removed from the committee because of financial COIs. It is not clear how often the NASEM removes provisional committee members for reasons of financial COIs.

The National Academy of Medicine has issued recommendations about conducting financial COI reviews that appear to differ from the NASEM’s approach, recommending that biomedical institutions create special conflict-of-interest committees to carry out reviews and noting that “…accountability is generally enhanced if public representatives serve on institutional panels that review individual relationships that may present conflicts of interest” [40].Such a committee dedicated to analyzing financial COIs does not appear to exist at the NASEM.

The NASEM’s prestigious journal, *Proceedings of the National Academy of Sciences* (*PNAS)*, also has different financial COI policies in place. For example, *PNAS* imposes sanctions on authors that “deliberately or recklessly failed to disclose conflicts of interest… including being banned from publishing in *PNAS* for a period of time” [53].

*A more conservative financial COI assessment*. It is noteworthy that several members of the committee that did not meet the financial COI criteria adopted in this analysis had ties to companies that a broader analysis may have cited as potentially introducing bias or imbalance. For example, one committee member directs a research project that accepts money from the Syngenta Foundation (associated with the agricultural biotechnology company Syngenta) and has recently published research that received in-kind contributions from agricultural biotechnology companies [54–57]. Another committee member serves as the director of a university-industry research center that receives money from an agricultural biotechnology company [58]. While these financial relationships may present potential financial COIs, they did not meet our strict criteria.

Similarly, two committee members we identified as having financial COIs, Richard Dixon and C. Neal Stewart, both have consulted extensively with agricultural biotechnology companies, but we did not identify this as a financial COI in Table 1 because we could not definitively document any consulting that met our criteria of having taken place within three years of the NASEM2016 report [59, 60].

In contrast, our search found no evidence of any committee members being financially associated with private entities that can be construed as having a financial interest in restricting or opposing genetic engineering. We found only one remote association.

One committee member serves as an unpaid advisor to a non-profit organic farm that has an apprenticeship program funded by the dairy industry [61,62]. The funders include Stonyfield, a company that promotes its avoidance of GE ingredients and that has played a prominent role in political campaigns to require labeling of food containing GE ingredients [63]. Another funder is the corporate foundation of Stonyfield’s parent company, Danone, which sells some products containing GE ingredients [64,65].

The various ties to industry described above did not meet our conservative definition of a financial COI, though a less conservative analysis may have viewed them differently. It is noteworthy that one public-interest group examining the NASEM2016 committee took a broader definition of COIs than our analysis took.

Citing The Academies’ requirements under FACA to form “fairly balanced” committees, that analysis identified financial COIs and also evidence of ideological positions that committee members had in favor of agricultural biotechnology in journal articles, statements to the press, or political advocacy work [66].

The NASEM’s COI policy notes: “An individual may have become committed to a fixed position on a particular issue through public statements…through publications…through close identification or association with the position or perspectives of a particular group, or through other personal or professional activities” [23].

The NASEM indicates that this would “ordinarily constitute a potential source of bias, but not a conflict of interest,” though it acknowledges it could, in some circumstances, present a COI [23].

Given the lack of clear definitions and guidelines on non-financial COIs from the NASEM or other scientific bodies, our analysis explicitly narrowed the focus to financial COI disclosure and did not consider committee members’ intellectual interests, professional associations, or public statements on biotechnology.

### Potential effects of COIs

The NASEM recruited a highly multi-disciplinary committee of scientists, including experts in wide-ranging fields like molecular biology, sociology, law and ecology for its NASEM2016 report. This array of expertise reflects the breadth of questions that the committee was asked to address in its report.

It is notable that the committee members we identified as having financial COIs comprised all of the committee’s expertise on key topics, including plant biotechnology, molecular biology, plant breeding, weed science and food science [67]. Presumably, committee members were asked to author the sections of the report relevant to their expertise, meaning entire chapters may have been written by committee members with financial COIs.

### Institutional COIs

Several recommendations around institutional COIs discuss establishing review boards to manage conflicts, but also note the difficulty forming review bodies that have both the independence and the power to effectively manage institutional conflicts [23, 68]. The NASEM differs from other academic institutions in that Congress has laid out specific requirements around financial COIs under FACA. These requirements do not currently speak to institutional COIs, though Congress has the power and independence to expand its requirements. Even the appearance of institutional COIs can affect the public perception of science.

## Conclusion

Our results show that The Academies failed to follow FACA requirements in making transparent the financial conflicts of interests of members of its Committee on Genetically Engineered Crops in its May 2016 publication. We also showed that the omitted disclosures may not have met the standards established by The Academies’ own guidelines or by contemporary standards of financial COI disclosure.

Among the limitations of this study are that we were able to identify financial COIs that were available in the open literature. We had no privileged sources. We also were not able to review all committee members’ research grants because most universities do not make these public. We were also not able to review committee member’s personal financial investments. As a result we may have missed some financial COIs.

Our analysis utilized slightly different criteria for assessing financial COIs than the NASEM, though five of the six committee members we identified as having financial COIs would appear to meet both the NASEM’s criteria and our own. Because the NASEM does not make public its internal financial COI reviews, we were limited in our ability to assess the organization’s rationale for not disclosing any of the financial COIs we identified. Likewise, because the NASEM shields from public review any public comments that stakeholders submit about financial COIs, we were limited in understanding the extent to which the NASEM was apprised to financial COIs from other sources. It is unclear why the NASEM does not make these public comments open for review, but, as noted, we located one public comment that provided the NASEM with documentation about several undisclosed financial COIs identified in this paper.

Between the financial COI disclosure forms submitted by committee members to the NASEM and the public comments the NASEM received, many or most of the financial COIs identified in our analysis should have been available for the NASEM to review.

Transparency of conflicts of interest is one of the foundational principles for ethical science because it gives readers a basis for drawing their own conclusions of bias and the confidence level of the paper or study [69]. The NASEM’s apparent failure to disclose financial COIs does not conform to best practices widely employed by the scientific community or to Congress’s requirements under FACA.

Disclosure is a first, critical step toward addressing the potential bias stemming from financial COIs. Removing potential bias from research also requires management of conflicts of interest, a topic that Congress partially addresses by requiring the NASEM to form “fairly balanced” committees of experts. While this issue is beyond the scope of this paper, it is noteworthy that every financial COI and institutional COI we identified in this analysis concerns financial interests related to private interests that favor the use of agricultural biotechnology, not companies with a financial interest in restricting the use of agricultural biotechnology.

Disclosure is not an elixir for preventing bias from financial COIs. The intent of FACA is to avoid financial COIs first and foremost, unless it is absolutely necessary to accept them. As Krimsky 2010 notes: “…unless transparency results in behavior change, it does not address the issues of bias and public trust” [6].

1. The NASEM, as one of the world’s most prestigious research institutions, is in a position to set a high standard for disclosure and management of financial COIs that other institutions can look to for guidance. By failing to disclose conflicts, The NASEM sends a message to the broader scientific community that its prestige is sufficient to forego rigorous standards of financial COI disclosure. A report as important and influential as *Genetically Engineered Crops*: *Past Experience and Future Prospects*, which may help shape federal rules and regulations around agricultural biotechnology, should aspire to the highest level of transparency in order to achieve the greatest public confidence in its objectivity.
2. In the light of our findings, we offer the following recommendations for improving the NASEM’s financial COI guidelines and their implementation.
3. Create a dedicated body within the NASEM to carry out financial COI reviews of committee members and board representatives, ideally with the assistance of public stakeholders to enhance accountability
4. Revise the NASEM’s COI policy to require committee members to submit three years of financial COI disclosures.
5. Eliminate the financial COI threshold of $10,000 for financial investments
6. Disclose all financial COIs of committee members that the NASEM deems unavoidable.
7. Make all public comments submitted to the NASEM committee part of the public record, as the federal government does in its policy making.
8. Disclose in reports any institutional funding the NASEM received in the previous three years from companies that could benefit from the NASEM’s findings.

Require the NASEM board representatives who have a financial interest in NASEM matters

to recuse themselves of any decisions regarding reports related to their financial interests.
